# Antenna design for microwave hepatic ablation using an axisymmetric electromagnetic model

**DOI:** 10.1186/1475-925X-5-15

**Published:** 2006-02-27

**Authors:** John M Bertram, Deshan Yang, Mark C Converse, John G Webster, David M Mahvi

**Affiliations:** 1 Biomedical Engineering, University of Wisconsin, Madison, WI 53706 USA; 2 Electrical and Computer Engineering, University of Wisconsin, Madison, WI 53706 USA; 3 Surgery, University of Wisconsin, Madison, WI 53792 USA

## Abstract

**Background:**

An axisymmetric finite element method (FEM) model was employed to demonstrate important techniques used in the design of antennas for hepatic microwave ablation (MWA). To effectively treat deep-seated hepatic tumors, these antennas should produce a highly localized specific absorption rate (SAR) pattern and be efficient radiators at approved generator frequencies.

**Methods and results:**

As an example, a double slot choked antenna for hepatic MWA was designed and implemented using FEMLAB™ 3.0.

**Discussion:**

This paper emphasizes the importance of factors that can affect simulation accuracy, which include boundary conditions, the dielectric properties of liver tissue, and mesh resolution.

## Introduction

Liver cancer is a significant worldwide public health issue. The disease has a mortality rate of 100% at 5 years in untreated cases [1] and results in the deaths of more than one million people each year worldwide [2-5]. Although liver cancer can be treated successfully by surgical resection of the malignant tissue, approximately 90% of patients with the disease are ineligible for the procedure due to factors such as insufficient hepatic reserve and the close proximity of tumors to blood vessels [1,6]. One promising alternative for these patients is hepatic microwave ablation (MWA), an experimental procedure in which an antenna is inserted percutaneously or during surgery [7] to induce cell necrosis through the heating of deep-seated tumors. Unlike other alternatives to resection such as RF ablation and cryoablation, MWA systems are able to produce large lesions in the presence of blood perfusion and are not restricted by tissue charring [5,7]. MWA also has favorable one, two, and three year survival rates of 96%, 83%, and 73% [8].

The many perceived advantages of microwave ablation have driven researchers to develop innovative antennas to effectively treat deep-seated, nonresectable hepatic tumors. These designs have focused largely on thin, coaxial-based interstitial antennas [9-11], which are minimally invasive and capable of delivering a large amount of electromagnetic power. These antennas can usually be classified as one of three types (dipole, slot, or monopole) based on their physical features and radiative properties.

To assist in antenna design for MWA, many researchers have employed the use of mathematical models rooted in computational electromagnetics (CEM), a discipline that employs numerical methods to describe propagation of electromagnetic waves. These methods center around the formulation of discrete solutions to the fundamental electromagnetic equations collectively referred to as Maxwell's equations. Three principal techniques exist within CEM. The first of these, the finite-difference time-domain (FDTD) method, is based on the Yee algorithm [12] and uses finite difference approximations of the time and space derivatives of Maxwell's curl equations to create a discrete three-dimensional representation of the electric and magnetic fields. This method has been widely used to numerically evaluate the electromagnetic radiation patterns of antennas in tissue [13-17], although long computation times are generally required. Another commonly used CEM technique is method of moments (MoM) [18,19], in which approximate numerical solutions to integral equations are formulated in the frequency domain to determine an unknown current distribution for an antenna. This distribution can then be subsequently extended to yield the antenna's radiation pattern.

An alternative to the two techniques above is the finite element method (FEM), which has been extensively used in simulations of cardiac and hepatic radio-frequency (RF) ablation [20,21]. FEM models can provide users with quick, accurate solutions to multiple systems of differential equations and as such, are well suited to heat transfer problems like ablation. In this study, we have adapted an existing axisymmetric electromagnetic FEM model that was implemented using FEMLAB™ 3.0 [22] to demonstrate proper techniques for the design of an antenna for hepatic MWA.

## Background

The FEM model used in this study was adapted from a coaxial slot antenna general model, developed by COMSOL for microwave cancer therapy [22]. In this model, the electric and magnetic fields associated with the time-varying transverse electromagnetic (TEM) wave generated by the microwave source propagating in a coaxial cable in the z-direction was expressed in 2D axially symmetric cylindrical coordinates as





with



where *r*_o _and *r*_1_are the outer and inner radii of the coaxial cable (m), *P*_in _is the input power (W), *ε*_rd _is the relative permittivity of the dielectric,  is the wave impedance in the dielectric of the coaxial cable (Ω),  is the intrinsic impedance (Ω), *ε*_0 _= 8.854 × 10^-12 ^is the permittivity of free space (F/m), *μ*_0 _= 4*π *× 10^-7 ^is the permeability of free space (H/m), ω = 2*π f *is the angular frequency (rad/s), *f *is the frequency (Hz), *k *= 2*π/λ *is the propagation constant (m^-1^), and *λ *is the wavelength (m). For interstitial coaxial-based antennas during MWA, the magnetic field is purely azimuthal. The electric field is in the radial direction only inside the coaxial cable and in both radial and the axial direction inside the tissue. This allows for the antenna to be modeled using an axisymmetric transverse magnetic (TM) wave formulation, in which the source was modeled as a low reflection boundary



with excitation magnetic field H_*φ*0 _= *C*/*Zr*. Such axisymmetric FEM models are highly desirable as they dramatically reduce computation time.

Careful examinations of both the antenna's specific absorption rate (SAR) pattern and frequency-dependent reflection coefficient in tissue are essential for the optimization of antennas for hepatic MWA. SAR represents the electromagnetic power deposited per unit mass in tissue (W/kg) and can be defined mathematically as



where *σ *is tissue conductivity (S/m) and *ρ *is tissue density (kg/m^3^) [23]. For the treatment of deep-seated hepatic tumors, the SAR pattern of an interstitial antenna should be highly localized near the distal tip of the antenna. Antenna efficiency can be quantified using the frequency-dependent reflection coefficient, which can be expressed logarithmically as



where *P*_r _indicates reflected power (W). The frequency where the reflection coefficient is minimum is commonly referred to as the resonant frequency and should be approximately the same as the operating frequency of the generator used. Antennas operating with high reflection coefficients (especially at higher power levels) can cause overheating of the feedline possibly leading to damage to the coaxial line or due to the thin outer conductor damage to the tissue [24].

**Figure 1:**
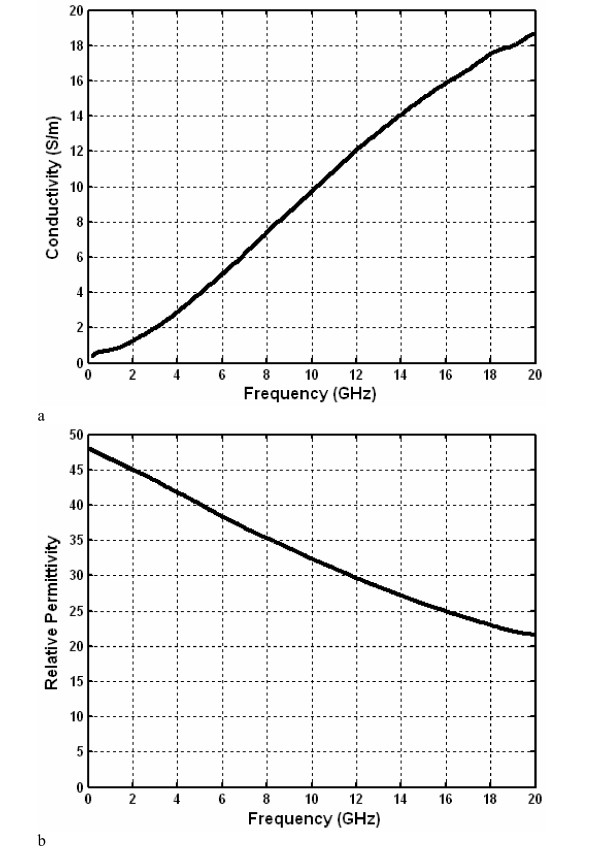
Dielectric properties of liver tissue measured ex-vivo on freshly excised bovine liver.

Critical to the development of electromagnetic models of hepatic MWA is an accurate knowledge of the dielectric properties of liver tissue. Fig. 1 shows the dielectric properties of fresh bovine liver that was obtained from a local slaughterhouse and measured approximately 1 h postslaughter. Measurements were obtained using the dielectric spectroscopy procedure described by Popovic *et al *[25], using a custom designed, stainless steel and glass borosilicate open-ended coaxial probe. These measurements were subsequently converted to complex permittivity using a rational function model [26,27].

## Materials and methods

### Antenna design

Several antenna designs have been shown to be effective for the treatment of deep-seated hepatic tumors. Recently, Saito *et al *[28] presented a design for a double slot antenna that is capable of achieving a higher degree of SAR localization than a standard single slot antenna and has been used in initial clinical trials. Another popular design, the cap-choke antenna [29-31], was originally designed for cardiac MWA applications but has been also found through independent investigation to be an effective applicator for hepatic MWA. In this design, the choke acts as a balun (which isolates the antenna and feedline to provide a balanced output) to prevent current backflow along the axial length of the antenna, resulting in high SAR localization near the distal tip of the antenna [32-34].

**Figure 2:**
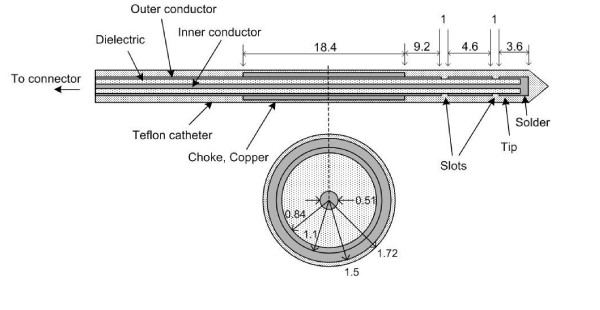
Axial and radial schematics of a coaxial-based double slot choked antenna designed for hepatic microwave ablation (MWA). All units are in mm.



Fig. 2 shows the general schematic for a double slot choked antenna. Antenna geometry parameters, the slot spacing, choke offset, choke length, etc, were chosen based on the effective wavelength in bovine liver tissue at 2.45 GHz, which was calculated using

where *c *is the speed of light in free space (m/s), *f *is the operating frequency of the microwave generator (2.45 GHz), and *ε*_r _= 44.4 is the relative permittivity of bovine liver tissue at the operating frequency; this yielded 18.4 mm for the effective wavelength. However, because the catheter and its thickness also affect the optimal geometry and performance of the antenna, equation 7 only provides a very crude approximation for the design. Fig. 2 shows that slot spacing, choke offset, and choke length correspond to 0.25*λ*_eff_, 0.5*λ*_eff_, and *λ*_eff _respectively, which were chosen to achieve localized power deposition near the distal tip of the antenna. Tip length and catheter thickness were adjusted to achieve resonance.

### Model development

For simplicity and to eliminate numerical error, the inner and outer conductors of the antenna were modeled using perfect electric conductor (PEC) boundary conditions. Low-reflecting boundary conditions were used along the model boundaries to prevent reflection artifacts and axial symmetry boundary conditions were also employed along the axis of rotation. Table 1 shows the frequency-independent material parameters used in this model. A modeling domain with radial and axial dimensions of 0.03 and 0.08 m was used for the simulation, and calculated results were plotted in terms of normalized SAR.

**Table 1:** Frequency-independent material parameters.

FEM Region	Material	Conductivity (S/m)	Relative Permittivity
Catheter	Teflon	0	2.1
Coaxial cable dielectric	PTFE	0	2.03

To determine the frequency response of the antenna, the model was simulated in FEMLAB™ at multiple discrete frequencies between 0.8 and 10 GHz using 50 MHz increments and the dielectric properties for bovine liver tissue shown in Fig. 1. At each frequency, a boundary integration of power outflow along the source was performed using FEMLAB™ to determine the net power delivered to the antenna. Subsequently, (6) was used to calculate the reflected power *P*_r _and frequency dependent reflection coefficient Γ(*f*).

To determine the optimal mesh resolution of the model and maximize computational efficiency, a convergence study was conducted in FEMLAB™ using the mesh parameters shown in Table 2. This was performed by gradually increasing mesh resolution along the effective source, catheter, and outer conductor boundaries of the antenna, as well as within the coaxial cable dielectric. Resolution in each of these regions was adjusted individually and numerical convergence resulted when a uniform change of less than 0.1% in the reflected power and normalized SAR was reached. As a result of the convergence study, maximum element size was set to 0.001 m along the catheter, outer conductor, and axial symmetry boundaries. For the source boundary and within the coaxial cable dielectric, maximum element size was also set to 0.0001 and 0.0002 m, respectively.

**Table 2:** FEMLAB™ mesh parameters.

Element growth rate	1.25
Maximum element size scaling factor	0.55
Mesh curvature cut off	0.0005
Mesh curvature factor	0.25

### Model validation

To validate the performance of the FEMLAB™ antenna model, a double slot choked antenna was constructed from UT-85 semirigid coaxial cable and gold-plated SMA connectors obtained from Microstock, Inc. This antenna was physically immersed in saline without a catheter and connected to an Agilent E8364A performance network analyzer (PNA) to measure the frequency-dependent reflection coefficient of the antenna. The dielectric properties of saline were measured using the same dielectric spectroscopy procedure mentioned above for liver tissue. These properties were subsequently used in a separate FEMLAB™ model to simulate the reflection coefficient of the antenna in saline without a catheter and were compared against measured results. A catheter was not used for the antenna in both measurement and FEMLAB model in order to avoid the possible effects by the catheter. Antenna performance is quite sensitive to the catheter thickness, but for practical reasons, the thickness could not be controlled exactly during the course of antenna construction. The comparison with the antenna without a catheter would still well serve the purpose to validate the computer model to experimental measurements.

## Results

**Figure 3:**
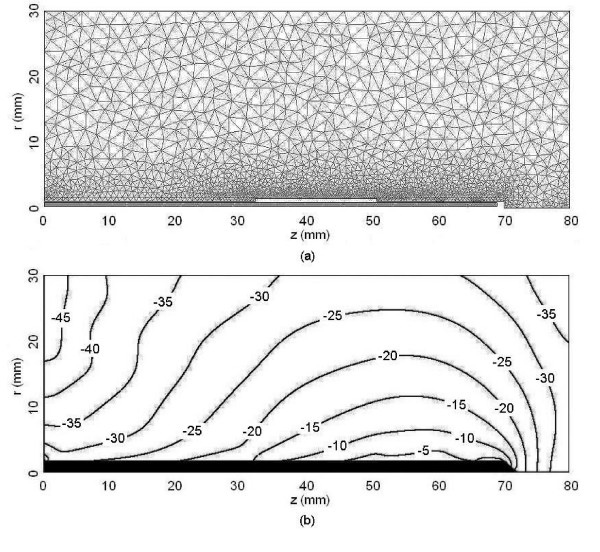
An axisymmetric electromagnetic model of the double slot choked antenna used for hepatic microwave ablation (MWA). (a) FEM model. (b) Logarithmic SAR distribution. Results were normalized using the maximum SAR obtained.

Fig. 3(a) shows the axisymmetric finite element mesh, which was generated by FEMLAB™ using the mesh parameters in Table 1 and the maximum element sizes determined by the convergence study. This mesh consists of 8920 triangular elements. Fig. 3(b) shows the corresponding two-dimensional normalized SAR distribution produced by FEMLAB™ for a double slot choked antenna. Calculation time for a single simulation on a personal computer with 2.8 GHz Pentium™ 4 processor and 1.5 GB memory required approximately 8 s. This same simulation required 75 s on a laptop computer with 1.6 GHz Pentium™ 4 processor and 512 MB memory.

**Figure 4:**
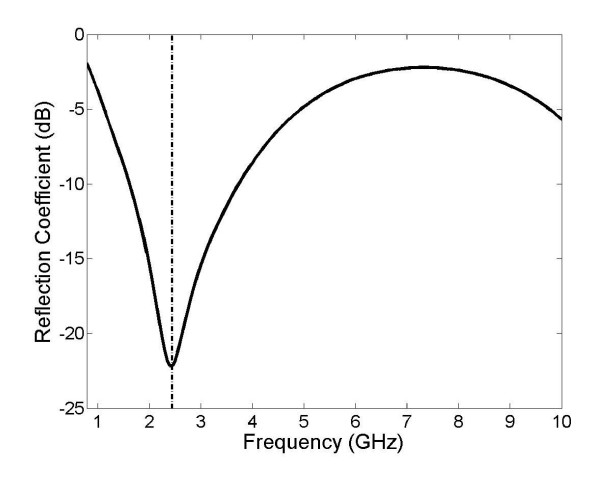
The simulated reflection coefficient of the double slot choked antenna, expressed logarithmically. The dashed line represents the commonly used MWA frequency of 2.45 GHz.

**Figure 5:**
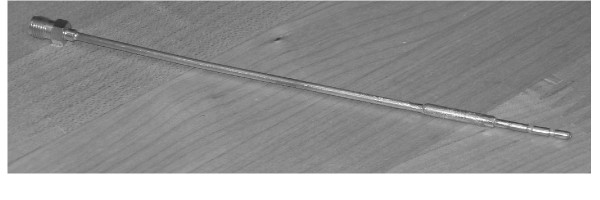
Photograph of the double slot choked antenna, shown without a catheter.

**Figure 6:**
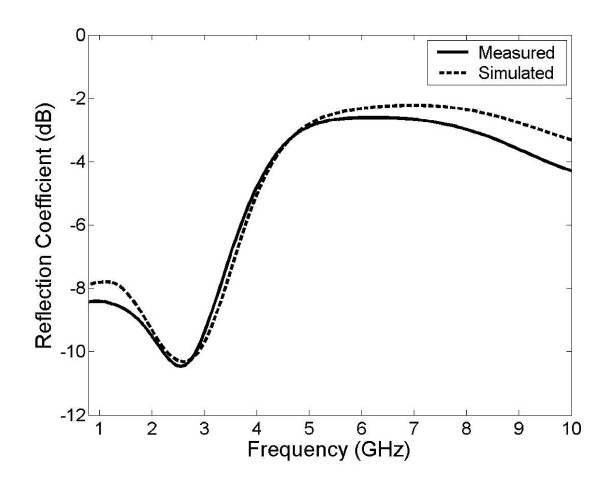
Comparison of simulated and experimental reflection coefficient of the double slot choked antenna, expressed logarithmically. The antenna was simulated and measured in saline rather than liver to ensure a homogenous medium for improved validation.

Fig. 4 shows the simulated antenna reflection coefficient in liver expressed logarithmically as a function of frequency with the dashed line indicating the commonly used MWA frequency of 2.45 GHz. This frequency response was calculated using the procedure described above and required approximately 30 min to complete. Fig. 5 shows the double slot choked antenna that was built to validate these results. Fig. 6 shows the simulated and measured reflection coefficient of this constructed antenna in saline.

## Discussion

Fig. 3(b) shows that the double slot choked antenna is able to provide a degree of power localization similar to those produced by other recently designed antennas. Volumes for 50% and 90% energy dissipation are 7.55 cm^3 ^and 99.3 cm^3 ^respectively. This initial analysis also shows that it may be capable of providing more localized power deposition than the earlier double slot antenna and standard cap-choke antenna, and should therefore be considered as a viable design for future research. Fig. 4 shows that the antenna is resonant at 2.45 GHz with a low reflection coefficient of approximately -22 dB. Reducing catheter thickness by 0.1 mm slightly improved localization but decreased the resonant frequency of the antenna by approximately 400 MHz. Such effects were expected because the catheter is an important part of the antenna structure. However more studies are apparently necessary to learn the exact effects of the catheter and the antenna geometry with catheter should be optimized together for the desired resonant frequency and SAR localization.

Several factors should be considered when designing and simulating antennas for hepatic MWA. Due to phase considerations, both antenna dimensions and the thickness of the catheter should be chosen and optimized based on the effective wavelength in tissue. As this quantity is highly dependent on the relative permittivity of the medium, it is also essential that electromagnetic models of antennas for hepatic MWA employ accurate dielectric properties of liver tissue. Great care should be taken when measuring these properties or using previously published data, as the dielectric properties of liver tissue are highly dispersive and change with tissue type [35], water content [36], and temperature [37].

In addition, tradeoffs that are not well understood exist between performance metrics such as SAR and the reflection coefficient. From our experience, it is usually more desirable to achieve a localized power deposition than a highly resonant antenna since external impedance matching networks can be used to improve antenna efficiency [38]. However, our additional experiments have shown that due to thermal conduction, a localized SAR pattern will only result in controlled tissue heating when treatment duration is minimized. Not only through the tissue, heat can also be effectively conducted by the antenna metal body and result in uncontrolled tissue heating along the antenna body for long treatment durations even with localized SAR pattern of the antenna.

Compared with other CEM software packages such as XFDTD™ and FEKO™, FEMLAB™ provides a user-friendly graphical interface for quick and reliable antenna design using axisymmetric electromagnetic models that can be integrated with MATLAB™ for increased functionality. Also, as the software was designed for multiphysics applications, it should also be ideal for future thermal simulations of hepatic MWA that are based on the Pennes bio-heat equation [39] and are dependent on both the electromagnetic and thermal properties of tissue. Initial work has also shown that electromagnetic models implemented using FEMLAB™ can be used in genetic algorithms [40] to better optimize antennas.

## Conclusion

An antenna for hepatic MWA was quickly and accurately simulated using an axisymmetric electromagnetic model implemented in FEMLAB™ 3.0. This model is ideal for analyzing the SAR patterns and reflection coefficient of antennas used in hepatic MWA, two metrics which are commonly used for the evaluation of such antennas. Using this model, a double slot choked antenna with performance comparable to current antennas was designed and evaluated.

## Authors' contributions

JB performed the research. All authors revised, read and approved the final manuscript.
